# A Systematic Review of Progress toward Unlocking the Power of Epigenetics in NSCLC: Latest Updates and Perspectives

**DOI:** 10.3390/cells12060905

**Published:** 2023-03-15

**Authors:** Anetta Sulewska, Lothar Pilz, Christian Manegold, Rodryg Ramlau, Radoslaw Charkiewicz, Jacek Niklinski

**Affiliations:** 1 Department of Clinical Molecular Biology, Medical University of Bialystok, 15-269 Bialystok, Poland; 2 Medical Faculty Mannheim, Heidelberg University, 68167 Mannheim, Germany; 3 Department of Oncology, Poznan University of Medical Sciences, 60-569 Poznan, Poland

**Keywords:** NSCLC, DNA methylation, miRNA, lncRNA, epigenetic therapy

## Abstract

Epigenetic research has the potential to improve our understanding of the pathogenesis of cancer, specifically non-small-cell lung cancer, and support our efforts to personalize the management of the disease. Epigenetic alterations are expected to have relevance for early detection, diagnosis, outcome prediction, and tumor response to therapy. Additionally, epi-drugs as therapeutic modalities may lead to the recovery of genes delaying tumor growth, thus increasing survival rates, and may be effective against tumors without druggable mutations. Epigenetic changes involve DNA methylation, histone modifications, and the activity of non-coding RNAs, causing gene expression changes and their mutual interactions. This systematic review, based on 110 studies, gives a comprehensive overview of new perspectives on diagnostic (28 studies) and prognostic (25 studies) epigenetic biomarkers, as well as epigenetic treatment options (57 studies) for non-small-cell lung cancer. This paper outlines the crosstalk between epigenetic and genetic factors as well as elucidates clinical contexts including epigenetic treatments, such as dietary supplements and food additives, which serve as anti-carcinogenic compounds and regulators of cellular epigenetics and which are used to reduce toxicity. Furthermore, a future-oriented exploration of epigenetic studies in NSCLC is presented. The findings suggest that additional studies are necessary to comprehend the mechanisms of epigenetic changes and investigate biomarkers, response rates, and tailored combinations of treatments. In the future, epigenetics could have the potential to become an integral part of diagnostics, prognostics, and personalized treatment in NSCLC.

## 1. Introduction

The research field of epigenetics has in recent years developed as an integral part of the diagnosis and treatment of oncological diseases. This review attempts to systematically demonstrate the already proven potential of epigenetics for the diagnosis and treatment of non-small-cell lung cancer (NSCLC). However, translating epigenetic findings into clinical practice is challenging due to the need to take into account various aspects and complexities of this research field. Findings such as contradictory and inconsistent results from a range of emerging studies complicate the selection of robust epigenetic biomarkers that can be used as diagnostic and prognostic tests and therapeutic targets.

Epigenetic modifications, which regulate gene expression without altering the DNA sequence, play a crucial role in the development and progression of NSCLC. The epigenetic landscape of NSCLC is characterized by a variety of alterations, including DNA methylation, histone modifications, and non-coding RNA expression [1,2].

DNA methylation is a well-known epigenetic alteration that involves the covalent addition of a methyl group to the cytosine residue of CpG dinucleotides, resulting in transcriptional repression. In NSCLC, aberrant DNA methylation patterns have been observed in tumor suppressor genes, oncogenes, and genes involved in cell cycle regulation and DNA repair. These epigenetic abnormalities can contribute to the development of NSCLC by promoting tumor cell proliferation, invasion, and metastasis [1,2].

Histone modifications, such as acetylation, methylation, phosphorylation, and ubiquitination, can also regulate gene expression by altering chromatin accessibility and gene transcription. In NSCLC, aberrant histone modifications have been implicated in the dysregulation of key signaling pathways, such as the PI3K/AKT/mTOR [3] and Wnt/β-catenin pathways [4]. Moreover, histone deacetylase (HDAC) inhibitors have been shown to have anticancer activity in NSCLC by inducing apoptosis and cell cycle arrest [5].

ncRNAs, such as microRNAs (miRNAs) and long non-coding RNAs (lncRNAs), have emerged as important regulators of gene expression in NSCLC. miRNAs can influence a wide range of biological processes, including cell proliferation, apoptosis, and differentiation, by binding to target mRNAs and inhibiting their translation or inducing their degradation. Dysregulation of miRNA expression has been shown to contribute to NSCLC tumorigenesis and progression. Similarly, lncRNAs can modulate gene expression by acting as decoys, scaffolds, or enhancers of chromatin-modifying enzymes. A growing body of evidence suggests that aberrant lncRNA expression is associated with NSCLC pathogenesis [6].

Epi-regulation involves interactions that are more complex than isolated events, such as the interaction between DNA methylation and miRNAs to inactivate protein-coding genes [7]. Notably, it has been revealed that more than 14% of all miRNA species are controlled via DNA methylation [8,9,10], and histone tails’ methylation has been proposed as an additional mechanism that influences miRNA genes [9,10,11]. Additionally, miRNAs can decrease the activity of long non-coding RNA (lncRNA) [12,13,14,15], and lncRNA can also be silenced by their histones’ deacetylation [15]. Furthermore, a set of epi-miRNA can indirectly target epigenetic regulators [16,17,18,19], while epigenetic modulators can directly interact with genetic changes. For example, DNA methylation is responsible for over 30% of disease-related germline point mutations [20].

Abnormalities in the epigenome landscape typically occur at the early stages of NSCLC, and these changes often modify as the disease progresses. Epi-drugs used as therapeutic modalities have the potential to lead to the recovery of affected genes, the restoration of genes that inhibit or delay tumor growth and survival, the abatement of the issue of tumor heterogeneity, and effectiveness against tumors without druggable mutations [2,21]. Furthermore, epigenetic drugs have the potential to re-sensitize cancer cells once they have become resistant to traditional or tyrosine kinase inhibitor (TKI) therapy. Combinations of pharmaceutics that target both genetic and epigenetic abnormalities are now being studied as being more powerful than those used to target only somatic mutations [21,22].

The epigenetic landscape of NSCLC has changed the perception of carcinogenesis, which was previously mainly based on the concept of malignant pathologies and genetic conditions. Consequently, it is now believed that carcinogenesis is a widely varying process influenced by multiple components in a number of steps of undefined length before it becomes clinically relevant. Advances in epigenetics have played an important role in improving our understanding of the underlying mechanisms, including RNA–RNA interactions and posttranscriptional regulations, which finally can result in the dysregulation of key oncogenes and tumor suppressor genes [23]. Thus, the processes of proliferation, invasion, metastasis, apoptosis, and cell cycle regulation will be affected, creating opportunities to detect cancer biomarkers for early cancer detection, prognostics, and therapeutic interventions. In lung cancer, hypermethylation comes along with *RASSF1A*, *MGMT*, *CDKN2A/p16*, and others. It was found out that lung adenocarcinoma links *HOXB9* DNA methylation to intrinsic EGFR-TKI resistance and leads to heterogenous outcomes [24].

Initial attempts at using combined targets to demonstrate a significant response in patients with lung cancer were described by Ansari et al., emphasizing the crucial role of hypomethylating agents, histone deacetylase (HDAC) inhibitors, and microRNA modulation [2]. Based on an improved understanding of the epigenetic landscape of NSCLC, clinical applications for targeting epigenetics in its treatment became viable with a variety of epigenetic regulators, such as DNMT, HDAC, and HDAC6 [25]. Other agents used in this therapy include azacytidine, entinostat, nivolumab, decitabine, and tetrahydrouridine [26]. More precise knowledge of the epigenetic landscape of immune-related diseases resulting from tobacco smoking will not only provide us with a comprehensive genomic map of the molecular changes induced by smoking but also enhance our understanding of its harm and relationship with diseases [27].

Our systematic review of epigenetics in non-small-cell lung cancer (NSCLC) provides an updated overview and fresh perspectives. We searched PubMed from inception to December 2022 for publications on the topic, focusing on (i) diagnostic epigenetic biomarkers in NSCLC, (ii) prognostic epigenetic biomarkers in NSCLC, and (iii) epigenetic-based therapy for NSCLC. We believe that epigenetic research will improve our understanding of the pathogenesis and lead to a more adapted management strategy for the disease.

## 2. Materials and Methods

### 2.1. Search Strategy

This study followed the Preferred Reporting Items for Systematic Reviews and Meta-analysis (PRISMA) 2020 statements checklist (Table 1) [28]. A literature search was conducted in PubMed as it is the most recommended database for systematic reviews due to its extensive range of articles, advanced search filters, and systematic search systems. Other databases such as Web of Science and Scopus may not provide as efficient and effective results for systematic literature reviews. Studies published from inception to 22 December 2022 were considered. To perform a comprehensive search, we used the following keywords and MeSH terms in different patterns: (“NSCLC”) AND (“diagnosis” OR “prognosis” OR “therapy” OR “DNA methylation” OR “histone modifications” OR “miRNA” OR “lncRNA” OR “exhaled breath condensate” OR “liquid biopsy”) (Table 2).

### 2.2. Eligibility Criteria

To be included in the review, studies had to meet the following criteria: (i) they had to be conducted on NSCLC patients or in NSCLC cell lines; (ii) they had to be aimed at diagnostic and/or prognostic epigenetic biomarkers and/or natural or synthetic epi-drugs; (iii) those conducted on humans had to provide related clinical pathological characteristics such as tumor stages of cancers (T), lymph node metastasis (LNM), or distant metastasis (DM); (iv) those with diagnostic value had to be limited to exhaled breath condensate, bronchial secretion, blood, or exosomes; (v) those with prognostic value had to contain information about survival outcomes; (vi) those with therapeutic value had to be limited to six phytochemicals (namely curcumin, magnolol, Chinese medicine Jinfukang, genistein, berberine, and cucurbitacin B) or six synthetic modalities (namely vorinostat, panobinostat, belinostat, trichostatin A, decitabine, and azacitidine); and (vii) they had to be published in English with the full-text paper had to be available. The search terms used in this systematic review are collated in Table 3.

Studies were excluded if one of the following existed: (i) duplicate publications; (ii) non-human studies, non-cell-line studies, animal studies, or studies not published in English; (iii) reviews, meta-analyses, case reports, letters, editorials, or expert opinions; (iv) studies without data available or with incomplete or retracted text.

### 2.3. Study Selection and Data Extraction

**Figure 1:**
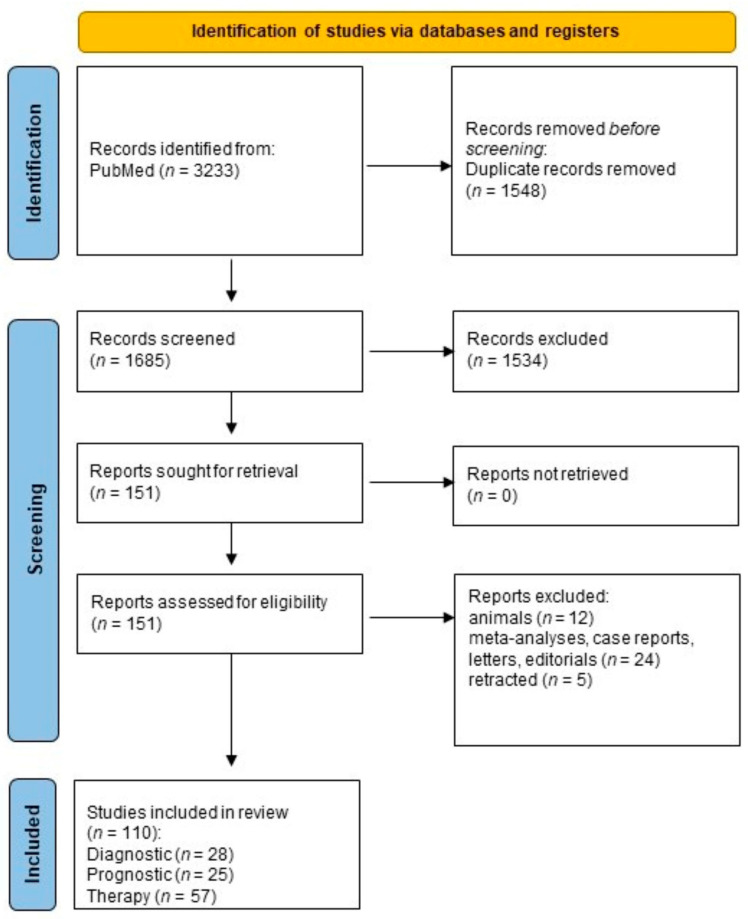
PRISMA 2020 flow diagram for systematic reviews including searches of databases and registers alone.

The selection process of publications complied with the inclusion criteria was performed manually by four independent authors (A.S., J.N., L.P., and C.M.), without the application of automation tools. After removing the duplicates, 1685 items were selected. We excluded 1534 citations by title and screened 151 abstracts for retrieval. Finally, 151 eligible studies were included. The queries and, consequently, the sections of the paper pertaining to diagnosis, prognosis, and therapy yielded 110 disjunctive studies that were used in three sections of the paper (Figure 1). Data were collected from the final 110 studies by four independent authors (A.S., J.N., L.P., and C.M.) without the utilization of automation. The extracted data included information on study design, population, biosource (cell cultures, tissues, biofluids), results, and conclusions.

For the main three topics investigated, namely, (i) diagnostic epigenetic biomarkers in NSCLC, (ii) prognostic epigenetic biomarkers in NSCLC, and (iii) epigenetic-based therapy for NSCLC, different methods for evaluating the results were used, which were (i) sensitivity and specificity/proportions, (ii) survival parameters, and (iii) tumor load, respectively. All authors judged the inclusion of the studies in the review based on these parameters (Table 4). Comparisons of the effects of the studies included are presented in Table 5 and in Section 4.2.1.

## 3. Results

The search terms used in this systematic review are collated in Table 3. For the main three topics investigated, namely, (I) diagnostic epigenetic biomarkers in NSCLC; (ii) prognostic epigenetic biomarkers in NSCLC; and (iii) epigenetic-based therapy for NSCLC, different approaches to evaluating the found results were used, such as (I) sensitivity and specificity/proportions; (ii) survival parameters; and (iii) tumor load, respectively. All authors judged the inclusion of the studies in the review based on these parameters (Table 4).

Although the application of methods varied for the studies subjected to the aforementioned three approach options, the independently judging authors only included studies where the measures described above were identifiable and explicit results were given. The differences in measures were outlined in the descriptions of protocols given in the respective subsections of each paper. Effects in these studies were given with confidence measures, if appropriate.

Comparisons of the effects in the studies were complicated due to the different conditions, markers, and agents used, which could mostly only be interpreted as qualitative results (see Table 5 and Section 4.2.1).

In terms of diagnostic epigenetic biomarkers in NSCLC, reported sensitivities and specificities varied significantly in exhaled breath condensate, bronchial secretions, and peripheral blood. This made it difficult to compare the diagnostic epigenetic biomarkers so that comparison is presented here simply as a description of facts.

**Figure 2:**
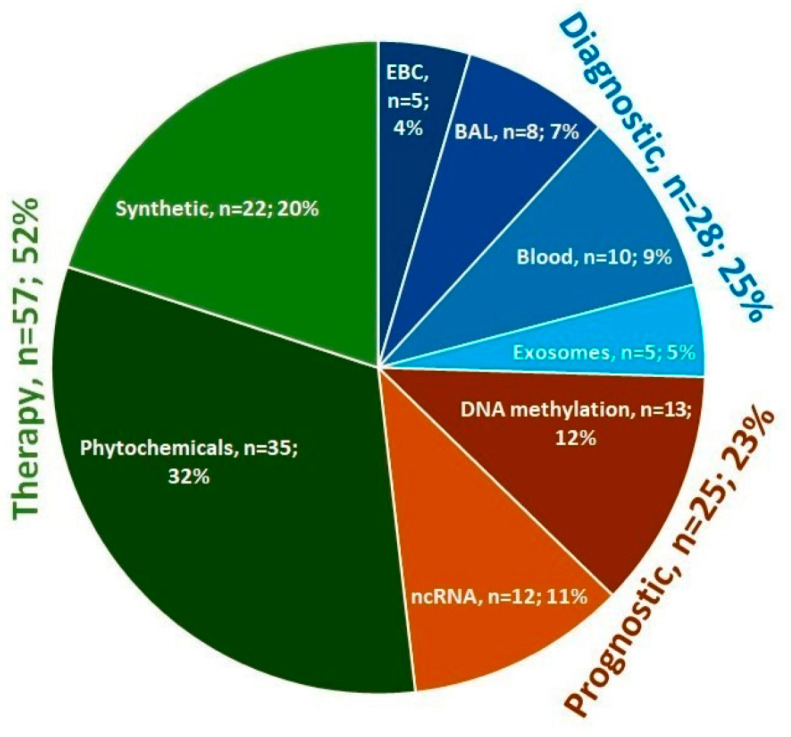
For the 110 studies in this review, the focus of research was in the following categories: diagnostics, *n* = 28 (BLUE with subgroups EBC, *n* = 5; BAL, *n* = 8; blood, *n* = 10; exosomes, *n* = 5); prognostics, *n* = 25 (RED with subgroups DNA methylation, *n* = 13; ncRNA, *n* = 12); group therapy, *n* = 57 (GREEN with subgropus phytochemicals, *n* = 35; synthetic, *n* = 22).

In the diagnostic area of research, there were 28 items, including 5 referring to exhaled breath condensate, 8 to bronchial secretion, 10 to blood, and 5 to exosomes; in the prognostic area, there were 25 items, with 13 referring to DNA methylation and 12 to non-coding RNA; and in the therapy area, there were 57 items, of which 35 referred to phytochemicals and 22 to synthetic epi-drugs. The diagnostic and prognostic areas of research had a comparable number of studies, whereas the therapy area was more extensive as there has been an increase in research on the use of both natural plant substances and synthetic pharmaceuticals for the treatment of NSCLC in recent years (Figure 1 and Figure 2).

The following section provided an interpretation of the data, addresses the consistency of results across studies, summarizes the findings, and concludes on the efficacy of diagnostic, prognostic, and therapeutic interventions. A brief discussion of pros and cons of the presented approaches is provided at the end of each subsection. Section 4.3.3 and Section 4.4 are not based on a systematically selected body of literature, but instead provide a broader elaboration on the prospects for the future.

## 4. Detailed Results and Discussion

### 4.1. Diagnostic Epigenetic Biomarkers in NSCLC

The lack of precise diagnostic methods for asymptomatic individuals limits the possibilities of early diagnosis of non-small-cell lung cancer (NSCLC). The first symptoms to manifest in an advanced stage of NSCLC are often unspecific and easily misattributed to other diseases. Chest radiographs and computed tomography (CT) scans expose patients to harmful radiation and can result in mistaking cancer with nonmalignant tumors. Furthermore, the histopathological examination, which is the cornerstone of the diagnosis, often has restrictions related to the size of biopsied samples, making some small specimens unsuitable for formalin-fixed paraffin-embedded processes [37].

Epigenetic evaluation can endorse and complement routine histology, especially when an unambiguous detection or subtyping of NSCLC is difficult. We have seen in our own studies that a panel of 14 lncRNA (*HAGLR*, *ADAMTS9-AS2*, *LINC00261*, *MCM3AP-AS1*, *TP53TG1*, *C14orf132*, *LINC00968*, *LINC00312*, *TP73-AS1*, *LOC344887*, *LINC00673*, *SOX2-OT*, *AFAP1-AS1*, *LOC730101*) can successfully differentiate cancerous from non-cancerous lung tissue (AUC value of 0.98 ± 0.01) as well as lung adenocarcinoma (LUAD) from lung squamous cell carcinoma (LUSC) (AUC value of 0.84 ± 0.09) [38]. However, these studies were conducted on tumor tissue obtained by invasive methods such as surgery, bronchoscopy, or imaging-guided biopsies, and, therefore, carried risks such as blood loss or clotting, pain, infection, or pneumonia. Furthermore, there may have been limitations on the range of patient’s condition, tumor heterogeneity, size, and location [39].

In our opinion, a real improvement would be to implement non-invasive procedures to collect stable and specific biomarkers [40,41]. In exhaled breath condensate (EBC) and biofluids, circulating tumor DNA (ctDNA), RNA (ctRNA), cells (CTCs), and extracellular vesicles (EVs) loaded with RNA, proteins, and DNA have been documented [42]. A detailed retrospective analysis is presented here to identify the possibility of non-invasive diagnostic methods for NSCLC detection in EBC and fluids such as exhaled breath condensate, bronchial secretion, blood, and related exosomes. We believe that these will be adopted in clinical practice if commercial tools are made available and easy to use.

#### 4.1.1. Exhaled Breath Condensate

Exhaled breath condensate (EBC) is obtained from the lungs and the lower part of the respiratory tract and is a mixture of volatile molecules and liquids secreted by mucous membranes. Thus, EBC is thought to provide molecular information on the development of lung cancer and is considered to be useful for the analysis of epigenetic aberrations [37].

It has been shown that miRNAs isolated from both serum and EBC may have similar patterns of expression and the potential to become diagnostic biomarkers for non-small-cell lung cancer (NSCLC). Chen et al. indicated that the expression level of *miRNA-21* in the blood serum and EBC in NSCLC patients was higher when compared with healthy controls. They also found the expression of the studied molecule intensified with the progression of the disease (stage I, II vs. stage III) and with the occurrence of metastases to lymph nodes [43]. Xie et al. noted that *miR-186* was downregulated in both serum and EBC of NSCLC patients, and this was linked to lymph node metastasis. Furthermore, they observed a connection between low expression of serum *miR-186* and higher serum carcinoembryonic antigen, C-reactive protein, and erythrocyte sedimentation rate [44]. Based on miRNAs from EBC, Perez-Sanchez et al. proposed two diagnostic signatures: the first for discrimination between lung cancer patients and healthy control groups (*miR-4507*, *miR-6777-5p*, and *miR-451*) and the second for recognition of LUAD from LUSC (*miR-4529-3p*, *miR-8075*, and *miR-7704*) [45]. Mozzoni et al. highlighted that deregulation of EBC *miR-21* and *miR-486* expression in NSCLC patients can be associated with any histological subtype, facilitating the detection of periphal-located adenocarcinomas, which are difficult to note using bronchoscopy or sputum cytology [46]. The utility of EBC in DNA methylation analysis was confirmed by Xiao et al., who focused on the methylation of *P16* in plasma, EBC, and tumors in NSCLC patients. They found that aberrant promoter methylation of *P16* was detected in 86.66% of tumors, 50% of blood plasma and 40% of EBC, while it was absent in the control group [47].

Exhaled breath condensate (EBC) has emerged as a promising diagnostic tool for NSCLC. Studies have demonstrated that miRNAs are frequently altered in the serum and EBC of NSCLC patients when compared to healthy controls. Moreover, aberrant promoter methylation of *P16* in tumors, blood plasma, and EBC has been reported. EBC can be obtained noninvasively with no discomfort to patients and thus has the potential to become a promising source of epigenetic biomarkers. However, we believe that there is a need for future standardization in sampling. A highly precise system for collecting respiratory droplets of lung-lining fluid is required to eliminate inter- and intra-individual variabilities and provide quantification of poorly expressed ncRNAs. Additionally, the collection system should be comprehensive enough to enable the detection of miRNAs with low abundancy, which are limited in serum and plasma, but sufficiently expressed in EBC. Consequently, established protocols for collecting, storing, and processing EBC are needed in order to reliably detect biomarkers for the diagnosis of NSCLC. In our opinion, widespread clinical use of these tools will only be made possible if the above-described pitfalls are overcome.

#### 4.1.2. Bronchial Secretions

The evaluation of epigenetic aberrations in bronchial secretions (sputum, bronchioalveolar lavage (BAL), also known as bronchioalveolar washing) facilitates screening of patients at risk of NSCLC. BAL reflects characteristics of the lung section, and due to its high content of epithelial cells, seems to be appropriate for biomarkers’ assessment [48].

Milares et al.’s comparison of the methylation of *DAPK*, *P16*, and *RASSF1A* in sputum and BAL did not show statistically significant differences between two bio sources. Discrepancies between the test and validation set were observed, which could have stemmed from false-positive results, presumably in the test group, or the usage of an inadequate method for validation [48]. Other authors have presented better results. Ma et al. recorded the methylation of *PCDHGB6*, *HOXA9,* and *RASSF1A* in tumor tissue and bronchial brushings with 92% sensitivity (AUC = 0.977, *p* < 0.001) and 80% specificity (AUC = 0.907, *p* < 0.001) [49]. Um et al. selected a panel of seven methylated genes (*TFAP2A*, *TBX15*, *PHF11*, *TOX2*, *PRR15*, *PDGFRA*, and *HOXA11*) from bronchial washing samples in genome-wide studies, discriminating cancerous from non-cancerous patients with 87% sensitivity and 83.3% specificity [50].

Not only was high precision in discriminating patients from healthy individuals achieved by analyses of one type of epigenetic change but also via a combination of different biomarkers. Su et al. noted that combined analyses of two miRNAs (*miR-31* and *miR-210*) and methylation of two genes in sputum (*RASSF1A* and *3OST2*) gave high sensitivity (87.3%) and specificity (90.3%) [51]. In another study, the same author and colleagues, using methylation-specific droplet digital PCR (ddMSP), selected four methylated genes (*HOXA9*, *RASSF1A*, *SOX17*, and *TAC1*) to create a logistic classifier that also successfully detected early stages of lung cancer (AUC 0.92; accuracy 88.8%; sensitivity 89.6%; specificity 90.6%) [52]. Studies of miRNAs by other authors have also been promising. Kim et al. presented how a five-miRNA signature (*miR-21*, *miR-143*, *miR-155*, *miR-210*, and *miR-372*) was detected in BAL with higher diagnostic sensitivity and specificity than in sputum (sensitivity 85.7% and 67.8%; specificity 100% and 90%, respectively) [53]. Rehbein et al. indicated that, in BAL, eight miRNAs (*miR 19b-1*, *1285*, *1289*, *1303*, *217*, *29a-5p*, *548-3p*, and *650*) discriminated between NSCLC and benign lung diseases [54]. Gupta et al. developed a biomarker panel of three sputum lncRNAs (*SNHG1*, *H19*, and *HOTAIR*), producing 82.09% sensitivity and 89.23% specificity for diagnosis of NSCLC [55].

In our view, the encouraging results create an opportunity for the future widespread use of bronchial washing and sputum in the diagnosis of NSCLC. As of yet, however, the applicability of epigenetic modifications as markers for these samples has not been completely assessed. While epigenetic analysis appears to have potential as a diagnostic tool, further research is necessary to validate its clinical usage in daily practice. Additionally, for greater accuracy and reproducibility, improvements to the procedures are also required. In our opinion, it is imperative to standardize the processing, uniformly validate the methodology, prevent contamination from normal or inflammatory cells, and increase the yield through enrichment of the bronchial epithelial cell fraction, and broad clinical use will only be made possible if the tools become easily available [50,53,54].

#### 4.1.3. Peripheral Blood

Peripheral blood is easier to collect and has a more extensive range of applications than bronchial washing. Furthermore, it is cost effective to use when screening asymptomatic individuals.

Montani et al. identified 13 serum miRNAs (miR-92a-3p, miR-30b-5p, miR-191-5p, miR-484, miR-328-3p, miR-30c-5p, miR-374a-5p, let-7d-5p, miR-331-3p, miR-29a-3p, miR-148a-3p, miR-223-3p, and miR-140-5p) which were able to assess the risk in both asymptomatic and symptomatic subjects, and distinguished benign from malignant lesions with 77.8% sensitivity and 74.8% specificity [56]. However, it is important to note that the epigenetic studies of different bio sources have not always yielded similar results. Hulbert et al. analyzed six methylated genes (SOX17, TAC1, HOXA7, CDO1, HOXA9, and ZFP42) to predict early-stage NSCLC (I-IIA). The best parameters were obtained for TAC1, HOXA7, and SOX17 in sputum (98% sensitivity and 71% specificity) and CDO1, TAC1, and SOX17 in plasma (93% sensitivity and 62% specificity) [57]. Conversely, Pu et al. selected three tissue and plasma-specific miRNAs (miR-211-3p, miR-3679-3p, and miR-4787-5p) that are typical for LUSC and three miRNAs (miR-3613-3p, miR-3675-3p, and miR-5571-5p) for LUAD [58]. Meanwhile, Ma et al. proposed using miRs-19b-3p and -29b-3p from peripheral blood mononuclear cells (PBMCs) to detect NSCLC (72.62% sensitivity and 82.61% specificity) and LUAD (80.00% sensitivity and 89.86% specificity) [59]. Not only do these analyses involve DNA methylation and miRNAs but also lncRNAs either separately or in combination with other factors. Tang et al. identified lncRNAs RP11-397D12.4, AC007403.1, and ERICH1-AS1, which were upregulated in the plasma of NSCLC patients and thus could be used to predict the onset of the disease [60]. Liang et al. suggested GAS5 circulating in the plasma of NSCLC patients as a potential diagnostic marker. When combined with carcinoembryonic antigen (CEA), they achieved an area under the ROC curve of 0.909 for the differentiation of NSCLC patients from healthy individuals [61]. Interestingly, Weber et al. observed that MALAT1 from a cellular fraction of peripheral blood was not suitable as a single diagnostic biomarker due to its low sensitivity (56%) [62]. To differentiate NSCLC patients from healthy individuals, three additional signatures comprising four serum miRNAs (miR-193b, miR-303, miR-141, and miR-200b) [63], two plasma miRNAs (miR-448 and miR-4478) [64], and two serum lncRNAs (XIST and HIF1A-AS1) [65] were proposed.

Bloodstream epigenetic studies involve serum, plasma, and PBMCs as the main sources of methylated DNA and ncRNAs. As previously noted, a broad range of molecules have been studied for the early detection and histological evaluation of NSCLC; however, there is still a need to reduce them to a reliable panel. Another limitation is the lack of standardized methodologies for nucleic acids’ isolation and quantification that can be easily applied in independent laboratories and generate reliable outcomes. We believe further studies are necessary to develop a robust biomarker panel with reliable and consistent results that can be easily implemented in clinical settings [56,57,58,60].

#### 4.1.4. Exosomes for Detection of NSCLC

Exosomes, endocytic vesicles secreted by both normal and malignant cells, can be found in liquid biopsies such as blood, urine, ascites, and cerebrospinal fluid [66], and evidence suggests they may have diagnostic, prognostic, and therapeutic significance, leading to extensive study of tumor-derived exosomes (TEXs) and their cargos [67].

Jin et al. identified 38 tumor-derived exosomal miRNAs that could be used to identify NSCLC, specifically LUAD or LUSC [68]. Cazzoli et al. developed a screening signature of three miRNAs (*miR-378a*, *miR-379*, *miR-139-5p*, and *miR-200b-5p*) and a diagnostic signature of six miRNAs (*miR-151a-5p*, *miR-30a-3p*, *miR-200b-5p*, *miR-629*, *miR-100*, and *miR-154-3p*) [69]. Furthermore, Lin et al. identified 640 differentially expressed lncRNAs in urinary exosomes of NSCLC (70 up- and 570 downregulated), which they suggested could be used as potential diagnostic biomarkers [70]. Additionally, Zang et al. observed the increased expression of lncRNA *UFC1* in serum exosomes, tumor tissue, and serum, which was linked to cell proliferation, migration, and invasion [71]. Moreover, Min et al. proposed that lncRNA *RP5-977B1*, which is overexpressed in serum exosomes, could serve as a useful diagnostic and prognostic biomarker of NSCLC (AUC 0.89) [72].

**Figure 3:**
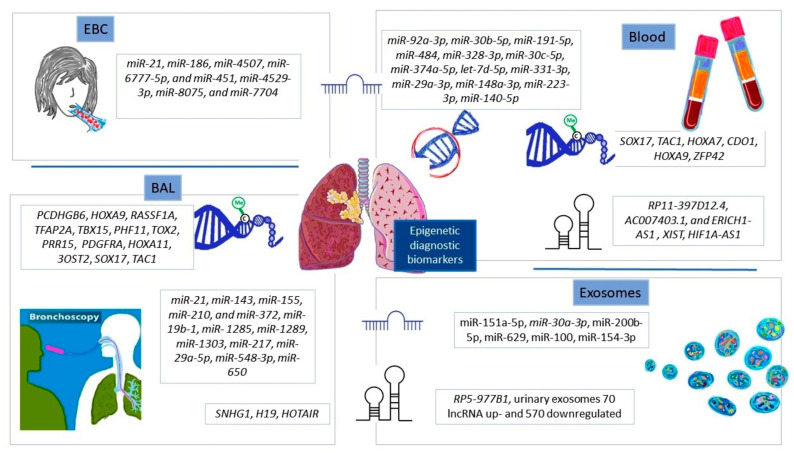
Epigenetic diagnostic biomarkers for NSCLC.

Exosomes, in addition to peripheral blood, are a promising source of highly sensitive and non-invasively collected epigenetic biomarkers. However, scientists still struggle with the complexity of biofluids containing other extracellular vesicles and the heterogeneity of exosomes themselves [73]. According to a total of 1254 studies from Vesiclepedia (December 2022), exosomes contain 349,988 proteins, 27,646 mRNA, 10,520 miRNAs, and 639 lipids (http://www.microvesicles.org, accessed on 22 December 2022) in 41 studied species. Navigating this multitude of molecules is not an easy task. There is also a need to standardize the isolation methods. Currently, immunoprecipitation, ultracentrifugation, sucrose density gradient ultracentrifugation, and commercial technologies are being used, but without a clear winner [73]. Moreover, the measurement of the size and concentration of the exosomes requires the application of nanoparticle tracking systems and transmission electron microscopy, which generate high costs, limiting accessibility for patients. To overcome these challenges and develop a satisfactory diagnostic test based on peripheral blood and exosomes in the near future, there is a need to increase the sensitivity and specificity of analyses, extend cohort studies, and perform independent validation. A summary of the epigenetic diagnostic biomarkers discussed in the paper is presented in Figure 3.

### 4.2. Prognostic Epigenetic Biomarkers in NSCLC

Determining the most efficacious course of treatment is essential for improving outcomes or even saving the lives of patients with NSCLC. Recent studies emphasize that the existing TNM staging system cannot accurately identify individuals who will benefit from adjuvant chemotherapy or targeted therapies [74]. In the era of precision medicine, genetic biomarkers such as *EGFR*, *ALK*, and *PD-L1* have already been incorporated into NSCLC prognostication [75]. Epigenetic biomarkers present extra opportunities, varying from singular epigenetic biomarkers that are adept at precisely classifying high-risk tumors, yet usually fail to recognize low-risk tumors, to intricate signatures which can surmount these impediments [76].

#### 4.2.1. From a Single Gene to Genome-Wide DNA Methylation Profiling

A substantial body of evidence referring to DNA methylation as a prognostic factor for NSCLC has been published. Despite the development of highly advanced technologies for screening the whole genome, there is still space for single-gene studies for fast, and low-cost evaluation of the methylation status of specific regions of the genome. However, the selection of prognostic factors based on single-gene methylation carries some challenges and constraints, which is discussed later in this paper.

Methylation of *DAPK1* [29] and *TUSC3* [30], in NSCLC tissue, was associated with improved overall survival (OS). However, in malignant pleural effusions of LUAD, the hypermethylation of *P16/INK4a* and *BRCA1* was linked with shorter survival, whereas hypermethylation of *RARβ* was linked with longer survival [31]. In addition, different classes of *HOXA* family genes were found to be downregulated in primary NSCLC tissues. The methylation of *HOXA2* and *HOXA10* was considered to be a negative prognostic factor in LUSC patients, though without clinical significance [32]. *HOXA9* methylation was associated with NSCLC in lifelong non-smokers with poor recurrence-free survival (RFS), after adjusting for clinicopathological characteristics. Despite this, the clinical significance of *HOXA9* hypermethylation at an early stage of LUSC has not yet been disclosed [33]. Zhang et al. were the first group to investigate the prognostic value of *PAX6* methylation in NSCLC [77]. Liu et al. showed that *TMEM196* hypermethylation was associated with shorter survival in TNM I–II LC patients, predicting the most aggressive and fatal disease [78]. Zhou et al. provided the first evidence for the methylation of *NPTX1* in NSCLC. In multivariate analysis models, the process was linked with shorter OS [34]. Furthermore, Sato et al. were the first to demonstrate that hypomethylation of *PTPRH* is related to a poor LUAD prognosis [35]. Xia et al. described for the first time the connection between downregulation via methylation of *miR-145* and poor differentiation, pleural invasion, advanced TNM staging, and lymph node metastasis in LUAD. They suggested that *miR-145* expression was both a prognostic marker and an identifier of locally advanced, surgically resected LUAD [79].

Single-gens methylation is associated with patients’ survival and TNM staging, suggesting the potential prognostic value for NSCLC. However, for official approval, additional independent studies are required to eliminate any lingering uncertainties, especially in terms of generated data. The lack of consistency between units can result from using different biological sources (tissues, biofluids) with possibly varying patterns of methylation or applying over-aggressive pretreatment of DNA. The latter scenario can cause partial bisulfite conversion of unmethylated cytosines and overestimate the methylation status of genes. To obtain repeatable and reliable results, there is a need to take into account both the unification of biological material for analyses and the handling of the bisulfite pretreatment carefully [30].

To access the whole landscape of DNA methylation, the application of high-throughput technologies generating large-scale data is unavoidable [80]. Then, when taking such a holistic approach to genome research, not only can the methylation picture be observed but also the relationship with other epigenetic and genetic factors can be monitored.

Lokk et al.’s complex studies revealed the hypermethylation of 496 CpGs (5′-cytosine-phosphate-guanine-3′) in 379 genes and hypomethylation of 373 CpGs in 335 genes for stage I NSCLC. Significantly, the validation narrowed the panel to 10 genes with prognostic value [81]. In addition, Kuo et al. found that methylation of eight genes—*AGTRL*, *ALDH1A3*, *BDKRB1*, *CTSE*, *EFNA2*, *NFAM1*, *SEMA4A*, and *TMEM129*—was linked to poor outcomes in early-stage LUAD, independent of Asian or Caucasian ethnicity [36]. Robles et al. then proposed a prognostic classifier for stage I LUAD, including genomic and epigenomic data. They found that *HOXA9* promoter methylation and expression of *BRCA1*, *XPO1*, *DLC1*, *HIF1α*, and *miR-21* were independently associated with the outcome, and combined results allowed for high-risk stage I patients’ detection [76]. Similarly, Bjaanæs et al. identified 33 highly methylated CpGs correlated with poor prognosis, the most methylated being the *HOXB* and *HOXC* clusters [82]. Comparisons of the effects of the included studies were complicated as the conditions, markers, and agents used were very different and could mostly only be qualified as qualitative results (Table 5).

Genomic screening technologies offer the potential to conduct genome-wide DNA methylation searches for new candidate prognostic biomarkers and explore the coexisting epigenetic and genetic abnormalities. Nevertheless, selecting the right high-throughput technology for global methylation analysis is a complex endeavor. Depending on the applied method, the commonly encountered restraints are as follows: significantly biased toward highly methylated areas, severely unrecognized CpG regions without restriction sites, substantially degraded DNA after bisulfite treatment, significantly restricted read number per run, and high error rates [83]. Therefore, to avoid hitting roadblocks or stalemates, when setting up a new study, substantial experimental and bioinformatics adjustments are required. As such, the authors believe that greater focus needs to be placed on the complexity and nuances of DNA methylation studies in order to yield accurate and reliable results for prognostic purposes. With careful selection of laboratory techniques and a well-defined research plan, it will be possible to find trustworthy prognostic markers that can provide insights into the clinical course for NSCLC.

#### 4.2.2. Non-Coding RNAs’ Expression Profiling

Dysregulation of non-coding RNAs (ncRNAs) observed from the onset to the advanced stage of disease can be strongly associated with clinicopathological features and predict survival outcomes, making them potential future prognostic factors for patients with NSCLC. Among ncRNAs, microRNAs (miRNAs) and long non-coding RNAs (lncRNAs) are the most powerful in terms of gene expression regulation. miRNAs are a subset of small non-coding RNAs, measuring an average of 22 nucleotides in length, while lncRNAs are longer, typically exceeding 200 nucleotides [84].

Liu et al. profiled 1105 miRNAs and selected *miR-29c* as a possible prognostic factor for LUAD. They observed that a decrease in *miR-29c* levels was associated with an unfavorable prognosis in stage IIIA (N2) LUAD [85]. Gao et al. identified seven miRNAs for predicting LUSC outcomes: high-risk *miR-139* and *miR-326* were negatively correlated with survival and upregulated in patients with high scores, while protective *miR-101-2, miR-182, miR-183, miR-190,* and *miR-944* were overexpressed in low-scoring patients [86]. Furthermore, the prognostic value for NSCLC could be determined by three plasma exosomal miRNA (*miR-23b-3p, miR-10b-5p,* and *miR-21-5p*) [87], four serum and tissue miRNA (*miR-1, miR-30d, miR-221*, and *miR-486*) [88], a newly discovered tissue and plasma *miR-1290* [89], and tissue *miR-424* [90].

The upregulation of lncRNA *HNF1A-AS1* has been associated with poor differentiation, advanced TNM stage, lymph node metastases, and shorter overall survival (OS) in LUAD [91], while the overexpression of *LCIIAR* has been positively correlated with immune infiltration of LUAD and poor survival rate [92]. Conversely, the downregulation of *BANCR* [15], *HMlincRNA717* [93], and *PANDAR* [94] have been linked to cancerogenesis and poor survival of NSCLC. Furthermore, nine highly sensitive and specific lncRNAs have been proposed for distinguishing relapsed and non-relapsed LUAD [95].

**Figure 4:**
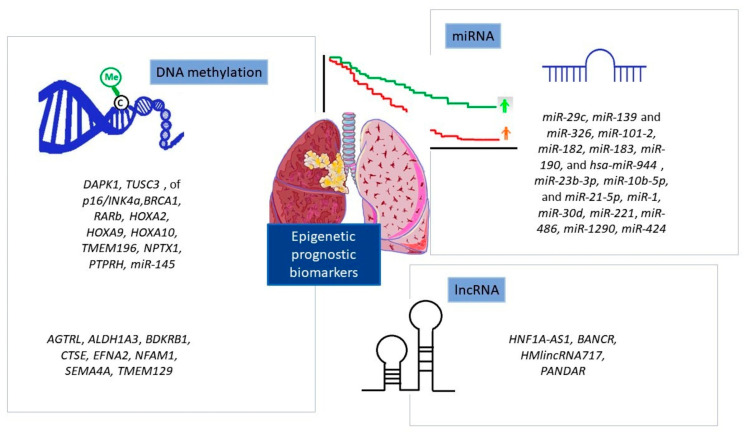
Epigenetic prognostic biomarkers for NSCLC.

Overall, ncRNAs’ overexpression is usually associated with poor OS, high TNM stage, and lymph node metastases; thus, their profiles may be potential prognosticators of the NSCLC outcome or even specific for particular subtypes. Nonetheless, further studies are needed to select the most powerful ncRNAs, and multi-institutional validation is crucial before making them suitable for prospective clinical trials. A summary of the epigenetic prognostic biomarkers discussed in this paper is presented in Figure 4.

### 4.3. Epigenetic-Based Therapy for NSCLC

The currently available therapies for NSCLC have shown varying efficacy, can increase the risk of drug resistance, and are only applicable to less than 50% of patients with targetable mutations [2]. Various natural and synthetic epigenetic modifiers have been investigated recently [96]. Epi-drugs have the potential to address the issue of tumor heterogeneity and, therefore, could be advantageous for patients without targetable mutations [97,98,99,100]. In our opinion, it is crucial to analyze the molecular basis and evaluate current research into these treatments, taking into account both the benefits and drawbacks, as well as the possibility of future utilization. Additionally, we think the implications for clinical practice and the potential for further investigation of epigenetic-based treatments should be explored.

#### 4.3.1. Natural Substances and Their Derivatives

Natural biologically derived compounds and their derivatives, which possess the capacity to modulate the epigenetic mechanisms of cancer cells, may constitute a novel generation of epi-drugs. This paper assesses the molecular basis and the efficacy in NSCLC treatment of six phytochemicals: curcumin, magnolol, Chinese medicine Jinfukang, genistein, berberine, and cucurbitacin B.

Curcumin (CU), a polyphenol extracted from the rhizome of Curcuma longa, has been identified as a promising chemosensitizer, radiosensitizer, and anticancer agent [101]. Ye et al. demonstrated that the administration of curcumin induces cytotoxic effects on NSCLC cells via a caspase-3-dependent mechanism, in which the *P53-miR-192-5p/215-XIAP* axis is engaged [102]. Similarly, Pan et al. observed that curcumin reduces proliferation, invasion, migration, and viability of NSCLC cells. Additionally, it increases the expression of *miR-142-5p* targeting *c-Myc* and inactivating the *Wnt/beta-catenin* signaling pathway [103]. He et al. confirmed that curcumin can sensitize NSCLC cells to crizotinib by restoring the expression of *miR-192-5p* [101]. Gao et al. revealed that curcumin-induced apoptosis can resensitize NSCLC cells to gemcitabine and upregulate lncRNA *MEG3* and *PTEN* [104]. Lastly, Wang et al. noted that curcumin inhibits the growth of NSCLC cells by downregulating lncRNA *UCA1* [105]. Further studies should be conducted to assess the potential of curcumin as an effective chemosensitizer and radiosensitizer for NSCLC. Additionally, it is important to understand how curcumin interacts with other treatments and its potential role in improving outcomes in patients with this type of cancer. If these studies prove successful, then curcumin may become a valuable addition to the currently available treatment options for NSCLC.

Magnolol (MG) and a polyphenol mixture derived from Magnolia officinalis were reported to have anti-cancer and anti-inflammatory properties. For example, Lee et al. reported that in NSCLC, magnolol was capable of promoting apoptotic signaling pathways and suppressing *STAT3/NF-κΒ*, epithelial–mesenchymal transition (EMT), and proteins involved in metastasis [106]. Zhao et al. likewise found that magnolol derivatives triggered G0/G1 phase cell cycle arrest, apoptosis, and autophagy in NSCLC cells [107]. Meanwhile, Tang et al. identified that compounds A13, C1, and C2 of magnolol derivatives exhibited the strongest anti-proliferative activities on NSCLC cells, causing efficient apoptosis in H1975 cells, as well as preventing the migration of HUVECs by inhibiting Cdk2, Cdk4, Cyclin E, and Cyclin D1, in addition to upregulating cleaved-PARP and cleaved-caspase 3 levels [108]. Moreover, Liu et al.’s findings showed that magnolol could not only influence the genetic makeup but also the epigenetic status of cells. They indicated that the compound significantly decreased the expression of class I histone deacetylases (HDACs), inducing cell apoptosis and activating pro-apoptotic signals (Bax, caspase3, TRAIL-R2) [109]. In this context, further study is needed to fully understand the potential of magnolol as an anti-cancer and anti-inflammatory agent.

Traditional Chinese medicine Jinfukang (JFK) is an oral liquid composed of 12 herbal extracts, which was approved by the China Food and Drug Administration in 1996 (Z19991043). It has since been widely used as an anti-NSCLC agent, with clinical effects of preventing metastasis, stabilizing tumor lesions, improving response, and extending patients’ survival; however, its biological function has yet to be fully elucidated [110]. Lu et al. indicated that JFK causes epigenetic alteration to H3K4Me3 of the promoters of tumor-related pathways’ genes such as *SUSD2*, *CCND2*, *BCL2A1*, and *TMEM158* [111]. Que et al. revealed that the application of the Chinese formula induced apoptosis of CTCs in NK cells through the *Fas/FasL* signaling pathway [112]; furthermore, subsequent studies showed that Jinfukang induces anoikis of CTCs by suppressing the *integrin/Src* axis [113], and inhibits metastasis of CTCs by suppressing the *EGF* pathway [114] or activating the ROS-mediated *ATM/ATR-P53* pathway and causing DNA damage [110]. Moreover, Huang et al. highlighted that the combination of Jin Fu Kang Decoction (JFKD) with gefitinib had a cytotoxic effect on gefitinib-resistant lung cancer cells [115]. Clinical trials are needed to investigate the efficacy of JFK and its combinations in the treatment of NSCLC in order to make recommendations for clinical practice.

Genistein (GNT), an isoflavone extracted from soybeans, has anti-inflammatory and pro-apoptotic activities, which diminish cancer cell growth, adhesion, and migration [116]. In terms of its epigenetic implications, research indicates that GNT enhances the *miRNA-37a* level [117] and demethylated *KEAP1* in A549 cells [118]. Similarly, a co-application with trichostatin A (TSA) (an HDAC inhibitor) in P53 wild A549 and H460 cells upregulates histone acetyltransferase (HAT), leading to increased histone H3/H4 acetylation [119]. In addition, genistein co-delivered with trichostatin A [119] or with *miRNA-29b* within aptamer-hybrid nanoparticles enriches apoptosis of NSCLC cells [120]. Various studies have further uncovered that GNT can serve as both a chemopreventive and a chemotherapeutic agent. Specifically, Xu et al. indicated that genistein, as a chemopreventive agent, activates *IMPDH2*, thus limiting the *PI3K/AKT/mTOR* pathway [116], while Zhang et al. demonstrated that genistein also acts as a chemotherapeutic agent and induces the *PI3K/AKT/HIF-1α* and *NF-κB/COX-2* signaling pathways [121]. In conclusion, genistein has diverse epigenetic and molecular activities that can be harnessed to inhibit cancer cell growth and apoptosis, both as a chemopreventive and a chemotherapeutic agent. Further research is needed to fully understand the potential of this natural compound in treating cancer.

Berberine (BBR) is an isoquinoline alkaloid found in crude extracts and decoctions of the bark, stems, and roots of various plants [122]. It is commercially available as a supplement for patients with diabetes and cardiovascular diseases [123], and its anti-cancer potential has been demonstrated through inducing cell cycle arrest, apoptosis, and autophagy; suppressing cell proliferation and invasion; and regulating miRNA and lncRNA expression and telomerase activity [122]. Kalaiarasi et al. observed that berberine, acting as an epigenetic factor, repressed histone deacetylase (HDAC) activity, while also downregulating four oncogenes (*TNF-α*, *COX-2*, *MMP-2*, and *MMP-9*), upregulating two tumor suppressor genes (*P21* and *P53*), actively regulating Bcl-2/Bax family proteins, and activating the caspase cascade apoptotic pathway [124]. Furthermore, Zheng et al. were the first researchers to identify that co-application of berberine with gefitinib regulated the inhibition of EMT stimulated by interactions between epi-molecules (*miR-34a-5p* and lncRNA *HOTAIR*) [125]. Likewise, Chen et al. demonstrated that BBR induced DNA damage and apoptosis of NSCLC through deregulation of the *Sin3A/TOP2B* pathway [126], as well as activation of the *ROS/ASK1/JNK* pathway [127]. Furthermore, Ni et al. suggested that berberine inhibited cell growth by dysregulating the expression of 646 genes, of which *RRM1, RRM2, LIG1,* and *POLE2* were downregulated and involved in DNA repair and replication [128]. Additionally, Liu et al. reported that demethyleneberberine (DMB) induced cell cycle arrest and cellular senescence by downregulating the *c-Myc/HIF-1α* pathway [129]. Finally, Alnuqaydan et al. observed that the incorporation of berberine–phytantriol-loaded liquid crystalline nanoparticles (BP-LCNs) into the A549 cell line resulted in the upregulation of *PTEN* and *P53* and downregulation of *KRT18* [123]. These findings suggest that berberine could potentially be an effective agent in cancer treatment. However, further clinical trials should be conducted to test the efficacy and safety of berberine as a potential therapeutic agent.

Cucurbitacin B (CuB) is a highly oxidized tetracyclic triterpenoid found in a wide range of plants, particularly within the Cucurbitaceae family. Its anti-inflammatory, antioxidant, and anti-cancer properties have been demonstrated through a range of mechanisms, such as epigenetic modifications and/or alterations to cellular pathways following treatment [130]. For example, Shukla et al. observed that CuB inhibited DNA methyltransferases and histone deacetylases in H1299 cells while reactivating two tumor suppressor genes (*CDKN1A* and *CDKN2A*), downregulating two oncogenes (*c-MYC* and *K-RAS*), and silencing the human telomerase reverse transcriptase gene (*hTERT*) [131]. Liu et al. demonstrated that CuB regulated cell proliferation and apoptosis by suppressing the *XIST/miR-let-7c/IL-6/STAT3* axis in NSCLC [132]. Similarly, Yu et al. showed that CuB reduced the proliferation of gefitinib-resistant PC9 cells by modulating the *miR-175p/STAT3* axis [133], while Yuan et al. highlighted the inhibition of epithelial–mesenchymal transition (EMT) in *TGF-β1*-induced A549 cells and gefitinib-resistant A549 cells via a decrease in ROS production and disruption of the *PI3K/Akt/mTOR* signaling pathway [134]. Furthermore, Liu et al. found that CuB suppressed the growth and invasion of gefitinib-resistant NSCLC cells by inducing lysosomal EGFR degradation and by downregulating the *CIP2A/PP2A/Akt* signaling axis [135]. Kusagawa et al. reported that CuB also downregulated *TNF-R1* at the initial stage of the TNF-α-dependent *NF-κB* signaling pathway [136], while Shukla et al. anticipated that CuB inhibited the metastasis of non-small-cell lung cancer (NSCLC) through suppression of the *Wnt/β-catenin* signaling axis [137]. Based on these findings, it is clear that CuB has the potential to be a powerful agent in the treatment of cancer. More research is needed to better understand the actions of CuB and its effects on different types of cancer.

Several studies have demonstrated the potential of phytochemicals to possess anticancer activity. These compounds have anti-inflammatory and antioxidant properties, which may inhibit cancerous cell proliferation [138]. As it is a natural and safe method of treatment, it may be beneficial for those who are unable to tolerate chemotherapy or radiation therapy. Certain phytochemicals can modify gene expression levels, potentially leading to alterations in tumor progression. As a result of their activity, either the upregulation of HAT or the downregulation of HDAC can be observed, and the expression of miRNA and lncRNA genes, responsible for tumor development and metastasis, can be modulated [103,105,139]. They can also modify the histones of the promoter of tumor-related pathways’ genes and directly affect the expression of oncogenes and tumor suppressor genes [104,106,111,116,124,130].

Clinical studies examining the safety and efficacy of plant-derived bioactive substances as treatments for non-small-cell lung cancer (NSCLC) have not yet—in our opinion—produced clear results. A potential issue is that these compounds are non-specific, meaning they may interact with other molecules in the body, potentially resulting in side effects. Nevertheless, plant compounds are often thought to be low in toxicity and have good accessibility, especially when incorporated into nanotechnology-based delivery systems. As such, there is still potential for their use in treating NSCLC in the future. Thus, further research and development could make them viable treatment options for NSCLC patients. The ability of herbal components to selectively target cancer cells or gene expression levels could make them particularly useful in addressing epigenetic alterations associated with NSCLC.

#### 4.3.2. Synthetic Epigenetic Modalities

There is ever-growing evidence that indicates synthetic epigenetic modalities may offer novel chances for tailored and effective treatment of NSCLC. Prominent examples in this regard include histone deacetylase inhibitors (HDACs) and DNA methyltransferase inhibitors (DNMTs). It has been observed that HDAC inhibitors, such as vorinostat (SAHA) [140], panobinostat (LBH589) [141], belinostat (PXD-101) [142], and trichostatin A (TSA) [143], can reduce the expression of genes associated with tumor growth and metastasis. Additionally, DNMT inhibitors, such as decitabine and azacitidine, have revealed their potential by reversing gene promoter hypermethylation, a hallmark of cancer [144]. The aforementioned epigenetic modalities can be utilized in conjunction with other epi-drugs or conventional treatments to improve outcomes for NSCLC patients.

Vorinostat (SAHA) has recently been investigated as a potential treatment for NSCLC through combination therapies. Takeuchi et al. reported promising results after combining vorinostat with gefitinib in patients with EGFR-mutated NSCLC who harbored a BIM deletion polymorphism [140]. Furthermore, the combination of vorinostat and pembrolizumab provides a safe and effective treatment for advanced NSCLC cases, as reported by Gray et al. [145]. Tu et al. found that co-delivery of vorinostat and simvastatin via a deformable liposome system (D-Lipo) increased their ability to infiltrate tumors, and consequently inhibited tumor growth [146]. Moreover, Takashina et al. observed enhanced therapeutic efficacy of combining vorinostat and 3-deazaneplanocin A, regardless of EGFR status. Furthermore, co-treatment with both drugs reduced histone H3 lysine 27 trimethylation and increased histone acetylation, depleted EZH2 and other PRC2 proteins, increased accumulation of p27Kip1, decreased cyclin A, and increased the apoptotic fraction [147]. Additionally, Liang et al. showed that a combination of azacitidine and vorinostat increased DNA accessibility and allowed transcription factors to bind more efficiently, resulting in the restoration of *PAX5* expression [148]. These findings indicate that epigenetic combinations might be leveraged to enhance therapeutic approaches for NSCLC. Further research is necessary to explore the complete therapeutic capability of these approaches and gain a more in-depth comprehension of their mechanisms of action in various subtypes of NSCLC. Moreover, additional investigations should be conducted to assess the efficacy and safety of epigenetic treatment for NSCLC, offering more data to assist with clinical decision-making.

Panobinostat (LBH589) has demonstrated effectiveness both in vitro and in vivo as a potential treatment option for NSCLC. Wang et al. discovered that combining panobinostat with chemotherapy, such as carboplatin, could increase its anti-tumor activity and thereby make this therapy an attractive choice for NSCLC [141]. Wu et al. identified lncRNA *GAS5-AS1* as a tumor suppressor in NSCLC, with its reduction linked to larger tumors, higher TNM stages, and lymph node metastasis. They noted that panobinostat with SAHA can upregulate *GAS5-AS1*’s expression, and its levels can be increased by specific knockdown of HDAC1 or HDAC3 [149]. Panobinostat in combination with radiotherapy or chemoradiotherapy for patients with inoperable stage III NSCLC may be an effective treatment, but there are restrictions associated with it. Takhar et al. demonstrated that administrated doses of up to 45 mg twice a week for RT and 20 mg twice a week for CRT are safe and effective with panobinostat, though there were two serious adverse events: rapid atrial fibrillation and tracheo-oesophageal fistula. Results showed an overall survival of 9 months, progression-free survival of 3 months, and a disease control rate of 66%. After 33 months of follow-up, all patients were still alive [150]. Further research is needed to fully assess the efficacy of panobinostat in treating NSCLC, including analyzing its long-term effects and the potential for adverse events. Additionally, research should be conducted to investigate possible treatment strategies for those cases in which panobinostat does not provide sufficient relief, as well as exploring alternative combination therapies.

Belinostat (PXD-101) is emerging as an effective drug for the treatment of NSCLC. To et al. showed that the combining of belinostat with cisplatin in platinum-resistant lung cancer cells had a synergistic cytotoxic effect, increased accumulation of cisplatin, and inhibited expression of *ABCC2* efflux transporter and *ERCC1* DNA repair gene. Additionally, a transcriptional repressor (negative cofactor 2) was observed to associate with the *ABCC2* promoter, implying that belinostat may be used as a drug resistance reversal agent when incorporated into chemotherapeutic regimens [142]. Furthermore, Ong et al. found that combining belinostat and CDK inhibitor seliciclib at clinically relevant concentrations was more effective than PXD101 alone in reducing cell proliferation and inducing apoptosis in NSCLC cells, with this effect being independent of the P53 status of the cells. Analysis of the apoptotic pathways suggested that caspase-mediated apoptosis plays a major role in this combination therapy [151]. Further research into belinostat’s potential as a cancer treatment is necessary to ascertain its efficacy and safety. To that end, clinical trials should be carried out to evaluate the effects of combining belinostat with other chemotherapeutic drugs in the treatment of NSCLC.

Trichostatin A (TSA) has been identified as a promising therapeutic option for NSCLC by multiple studies. Erkin et al. determined that trichostatin A, pracinostat, TGX-221, PHA-793887, AG-879, and IMD0354 had the potential to reverse the expression of the differentially expressed genes (DEG) genes such as *CDC20*, *AURKA*, *CDK1*, *EZH2*, and *CDKN2A* in NSCLC [143]. Sindo et al. suggested that quisinostat (JNJ-2648158) and TSA can induce G1 arrest and inhibit migration of A549 cells, as well as improve mitochondrial respiration and elevate *CLDN-7* expression. The combination of JQ1 and TSA has been shown to be more effective in inhibiting growth in H1975-OR and H1975-P cells than either substance alone, as well as inhibiting simertinib-resistant non-small-cell lung cancer [152]. Lastly, Tang et al. indicated that TSA may be a potential therapeutic option in lung cancer patients with high *IGFBP2* expression as it could reverse chemoresistance and enhance autophagy [153]. Consequently, these studies suggest that quisinostat, TSA, and their combination may be potential therapeutic targets for NSCLC as they can stop cancer cell growth, reverse chemoresistance, and improve mitochondrial respiration.

DNMT inhibitors, such as azacitidine (5-azacytidine) and decitabine (5′-aza-2′ deoxycytidine), have demonstrated good tolerability in NSCLC patients with evidence of partial responses and stable disease. Evidence of good tolerability of azacitidine was found in NSCLC patients following aerosolized treatment; a partial response was observed in one of eight patients, with two having stable disease and detectable plasma levels of azacitidine. Pre- and post-treatment bronchoscopy revealed a decrease in global DNA methylation of the bronchial epithelium, suggesting non-cytotoxic doses of inhaled azacitidine may be effective in treating malignant and/or premalignant lung lesions [154]. A synthetic lethal interaction between *TMPRSS4* and *DDR1* was identified as a novel vulnerability in NSCLC, with hypomethylation of the *DDR1* promoter found to be an independent prognostic factor and 5-azacitidine treatment increasing *DDR1* levels. Cells lacking *TMPRSS4* were highly sensitive to the DDR inhibitor dasatinib and to cisplatin after double knock-down [155]. Additionally, Nehme et al. revealed that the *TBX2* subfamily of transcription factors—suppressed in NSCLC—was significantly induced by 5-azacitidine [156]. Beyond that, 5-azacitidine was also demonstrated to be involved in the modulation of *hOGG1* expression levels in NSCLC tissues, with its expression reduced in NSCLC tissues and with methylation of the +322–327 CpG site in the 5′-UTR region hindering the recruitment of Sp1 to the 5′-UTR of *hOGG1*. Treatment with 5-azacitidine was able to restore *hOGG1* expression levels in both cell lines and tissues [157].

Similarly, demethylation of *SFRP2* has been linked to suppressed NSCLC invasion, with decreased ZEB1 and MMP9 levels in NSCLC cell lines observed on treatment with 5′-aza-deoxycytidine (decitabine), another demethylation factor [158]. Furthermore, combining aza-deoxycytidine with HDAC has shown promise as a treatment for NSCLC. Pre-clinical models have indicated altered localization and functional status of immune cells, suppressed angiogenic potential, and increased CCL5. Patients with MYC-high, CCL5-low tumors may respond best to this epigenetic therapy, and its efficacy is currently being tested in a clinical trial [159]. Decitabine and aspirin have been demonstrated to be an effective approach to NSCLC treatment, inhibiting tumor cell growth and metastasis by hampering the *β-catenin/STAT3* signaling pathway and significantly reducing tumor growth compared to single-agent treatment or the control [160]. Additionally, epigenetic regulation of the *miR-200/ZEB* axis has been identified as a major factor in TGF-β1-induced EMT in PC9 cells, and decitabine has been suggested as a potential therapeutic strategy to prevent tumor development, reversing TGF-β1-induced EMT in PC9 cells by sparking epigenetic changes to the *miR-200* family [161]. Furthermore, 5-Aza-2′-deoxycytidine and trichostatin A have been suggested to epigenetically restore *CTGF* expression in NSCLC, with their expression significantly diminished in NSCLC tissues [162].

**Figure 5:**
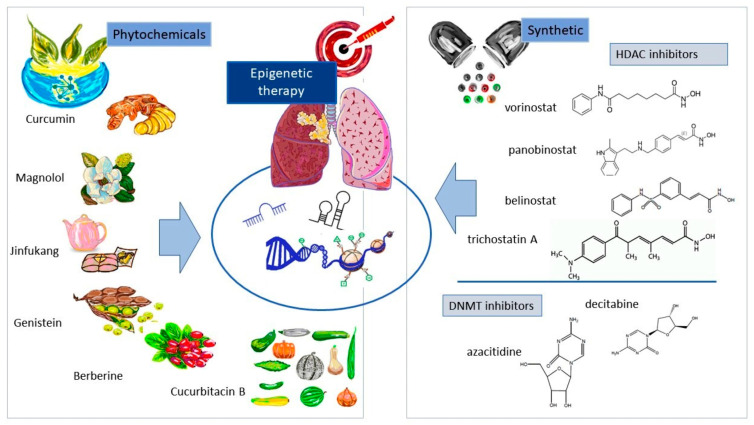
Epigenetic-based therapy for NSCLC.

Epigenetic modalities, such as histone deacetylase inhibitors (HDACs) and DNA methyltransferase inhibitors (DNMTs), may provide personalized and effective treatment options for non-small-cell lung cancer (NSCLC). Vorinostat, panobinostat, belinostat, decitabine, and azacitidine have been studied in combination with other drugs such as gefitinib, pembrolizumab, simvastatin, 3-deazaneplanocin A, CDK inhibitors, chemotherapies, MEK inhibitors, and radiological treatments. These combinations may alter gene expression and epigenetic pathways, including promoter hypermethylation, histone modification, and ncRNA expression, leading to improved therapeutic efficacy and tumor-suppressive effects, with an increased apoptotic fraction and decreased cell proliferation observed in many studies. Additionally, these epigenetic combinations may potentially be employed to better inform clinical decision-making and optimize therapeutic strategies for NSCLC patients with different subtypes. A summary of the epigenetic-based therapy options discussed in this paper is presented in Figure 5.

#### 4.3.3. Perspectives on Epigenetic Therapy for NSCLC

Epigenetic therapy refers to treatments that alter gene expression and function in order to combat disease. In the context of NSCLC, epigenetic therapies seek to target the genetic alterations associated with the cancer in order to reduce tumor burden, inhibit tumor progression, and improve clinical outcomes. These therapies focus on modifying gene expression by targeting epigenetic regulators such as histone modification enzymes, DNA methyltransferases, and histone demethylases [2]. Epigenetic therapies can also be delivered via small molecules or gene therapy [146,163]. Currently, epigenetic therapies are being studied in combination with existing treatments such as chemotherapy, radiotherapy, immunotherapy, and surgical resection to enhance the efficacy of these therapies and to increase survival rates [101,141,142,164].

However, scientific explanations for the effectiveness of combined therapy in NSCLC are contradictory. One hypothesis is that combining different treatment modalities can overcome the resistance that cancer cells develop to individual therapies. For example, BET inhibitors can target epigenetic regulators, while chemotherapy can target rapidly dividing cells. Combining these two modalities can address different aspects of cancer and improve overall response rates [165]. Another hypothesis is that combining treatment modalities can enhance the immune response to cancer. For example, epigenetic therapy can stimulate immune cell function, making the immune cells more effective at attacking cancer cells [164]. Despite this, researchers argue that the effectiveness of each treatment option may vary depending on the stage of cancer and the patient’s overall health, and the benefits may come at the cost of increased toxicity and side effects [101,141,142,164].

The main challenge facing researchers is the development of novel epigenetic or combined therapies that are effective, safe, and well tolerated. A key obstacle is the lack of robust biomarkers for assessing the efficacy of epigenetic interventions. In NSCLC, it is difficult to determine whether epigenetic therapies are having any impact on outcomes due to the inherent heterogeneity of the disease [1]. Additionally, the effects of epigenetic therapies can vary significantly between individuals and even within the same patient over time [2]. Therefore, identifying reliable biomarkers to measure the efficacy of these therapies is critical for advancing research in this area.

In addition to developing reliable biomarkers, researchers must also consider the effects of epigenetic or combined therapies on healthy cells and tissues. Some epigenetic and combined therapies may lead to unwanted side effects or an increased risk of toxicity, so researchers must identify methods of selectively targeting cancer cells without affecting normal cells. Additionally, researchers must develop tools to assess long-term safety and efficacy as some epigenetic therapies may have delayed side effects or unforeseen consequences [123,145,146,150,151,154].

The opportunities presented by epigenetic and combined therapies include the potential to personalize treatments and repurpose existing drugs for NSCLC based on genetic profiling or the cancer characteristics. In this way, treatments could be formulated to target specific mutations or epigenetic alterations associated with NSCLC. Furthermore, existing drugs that are safe and effective in other contexts could be repurposed for use in NSCLC by targeting epigenetic regulators.

In our opinion, epigenetic therapy presents an exciting opportunity for treating NSCLC. However, further research is needed to identify reliable biomarkers for assessing efficacy and to develop methods of selectively targeting cancer cells. Additionally, researchers must consider the effects of this therapy on healthy cells and tissues, and develop tools to assess long-term safety and efficacy. Through such research, epigenetic therapy could potentially transform the way NSCLC is treated, providing patients with personalized treatments that are safe, effective, and tailored to their individual needs.

The future of epigenetic therapy for NSCLC appears to be promising, with a wide range of potential opportunities on the horizon. With the development of new biomarkers, better methods of selectively targeting cancer cells, and more comprehensive assessments of safety and efficacy, epigenetic therapy could become a powerful tool for combating this type of cancer.

### 4.4. Summary and Future Strategies for Epigenetic Research on NSCLC

Epigenetics is a rapidly advancing field of research in the medical community and its relevance to NSCLC is becoming increasingly evident. Epigenetic mechanisms are known to play an important role in the regulation of gene expression, potentially leading to the development of various types of cancer [1,2].

The key areas of focus for future investigation include understanding the impact of epigenetic mechanisms on disease initiation and progression, identifying novel epigenetic markers for early detection, exploring epigenetic-based treatment options, assessing epigenetic variability across individuals, applying high-throughput technologies and AI in research, and examining the implications of epigenetics for health.

Firstly, much research will be required to understand the epigenetic mechanisms that are involved in the initiation and progression of NSCLC. This is because epigenetic processes, such as DNA methylation, histone modifications, and abnormal expression of miRNAs and lncRNAs are primary contributors to protein-coding gene expression and can have direct implications for the development and progression of cancer [7,8,11,12].

Secondly, novel epigenetic markers may also be identified to facilitate early detection of NSCLC. Increasing evidence suggests that specific epigenetic markers appear to be closely related to the stage of tumor development and are able to provide useful diagnostic information. For instance, some studies have demonstrated that tumor suppressor genes may be reactivated by epigenetic modifications, such as histone acetylation, thus providing an indirect mechanism for the early detection of cancer [22]. Furthermore, recent advances in next-generation sequencing technologies, such as targeted methyl sequencing, miRNA-seq, and RNA-seq, have made it possible to detect specific epigenetic markers at a much higher resolution [32,68,166]. This could potentially guide the development of targeted assays with improved predictive accuracy for early NSCLC detection.

Thirdly, epigenetic-based treatments will also be explored as potential therapeutic options for NSCLC. As mentioned previously, epigenetic modifications can profoundly influence gene expression and, thus, can be used to regulate the activity of many cellular pathways [97,98]. For instance, two classes of drugs, DNA methyltransferase inhibitors and histone deacetylase inhibitors, are being studied as potential therapeutic options for some types of cancer. It is hypothesized that these medications may be able to reverse epigenetic modifications that are associated with cancer progression and thus help to slow tumor growth and improve survival outcomes [154,155,160].

Fourthly, epigenetic variability between individuals will also be an important area of focus for future research. This is because genetic variability has been used to explain inter-individual differences in the risk of developing certain diseases, including cancer. However, recent studies have suggested that epigenetic modifications can also influence disease risk and progression, highlighting the need to further understand this epigenetic variability. One promising approach is comparison of epigenetic profiles in healthy individuals versus those with NSCLC. This may help to identify epigenetic markers that can indicate a predisposition to the condition [76].

Fifthly, high-throughput technologies and artificial intelligence (AI) have been identified as powerful tools for the analysis of large-scale epigenomic datasets in non-small-cell lung cancer (NSCLC). These technologies enable comprehensive screening of various epigenetic alterations such as histone modification, DNA methylation, chromatin organization, and miRNA and lncRNA expression, which can be used to identify signatures associated with disease progression, therapeutic response, and drug sensitivity [167,168]. AI provides rapid and efficient assessments of the effects of environmental and genetic factors on NSCLC progression and can generate predictive models. By combining AI and high-throughput technologies for epigenomic studies, a better understanding of the molecular mechanisms underlying this disease can be achieved, which could lead to the development of novel targeted therapies [167,169].

Finally, public epigenetic studies of NSCLC will rapidly become an important part of personalized medicine, offering potential insights that can lead to improved outcomes and quality of life for patients. By learning more about the mechanisms governing disease progression, clinicians can better tailor treatment plans according to individual patients’ needs and provide more effective preventative health strategies. These data can also be used to develop tailored lifestyle interventions, develop new treatments and biomarkers, and inform healthcare providers and policymakers [170]. However, in order to ensure the ethical use of these data, it is important to put in place guidelines and regulations that protect individuals’ autonomy and privacy. By taking these steps and continuing to study the impact of epigenetics on NSCLC and other types of cancer, researchers and healthcare providers may be able to improve outcomes for patients and reduce the burden of disease on the population as a whole [170,171].

## 5. Conclusions

In conclusion, this paper has provided a comprehensive overview of the latest updates and perspectives on epigenetics in NSCLC, with 110 original studies covering diagnostic, prognostic, and therapeutic areas. The results indicate that, given the growing research and advancements, epigenetic mechanisms may have a substantial influence on this aggressive form of cancer. Consequently, we conclude that further investigation is required to ascertain the underlying mechanisms and improve therapeutic outcomes. Specifically, queries such as how to develop better biomarkers for early detection, optimize patient response rates, and use combination therapies to achieve personalized treatments need to be addressed in order to capitalize on the potential of epigenetic therapies. Ultimately, when used correctly, epigenetics may offer upgraded sensitivity to the molecular pathways of cancer and provide more efficacious personalized treatments for NSCLC patients. To optimize the potential of epigenetic therapies, concerted efforts must be made by clinicians and researchers to advocate for evidence-based research and integration of the findings into clinical practice, as well as the development of national and international guidelines, and an organized plan to foster collaboration among clinicians, scientists, and industry stakeholders.

## Figures and Tables

**Table 1:** PRISMA 2020 main checklist.

Topic	No.	Item	Location Where Item Is Reported
TITLE			
Title	1	Identify the report as a systematic review.	page 1
ABSTRACT			
Abstract	2	See PRISMA 2020 for Abstracts checklist.	
INTRODUCTION			
Rationale	3	Describe the rationale for the review in the context of existing knowledge.	pages 2 and 3
Objectives	4	Provide an explicit statement of the objective(s) or question(s) the review addresses.	page 3
METHODS			
Eligibility criteria	5	Specify the inclusion and exclusion criteria for the review and how studies were grouped for the syntheses.	page 7
Information sources	6	Specify all databases, registers, websites, organizations, reference lists, and other sources searched or consulted to identify studies. Specify the date when each source was last searched or consulted.	page 3
Search strategy	7	Present the full search strategies for all databases, registers, and websites, including any filters and limits used.	pages 6 and 7
Selection process	8	Specify the methods used to decide whether a study met the inclusion criteria of the review, including how many reviewers screened each record and each report retrieved, whether they worked independently, and if applicable, details of automation tools used in the process.	pages 7 and 8
Data collection process	9	Specify the methods used to collect data from reports, including how many reviewers collected data from each report, whether they worked independently, any processes for obtaining or confirming data from study investigators, and if applicable, details of automation tools used in the process.	pages 7 and 8
Data items	10a	List and define all outcomes for which data were sought. Specify whether all results that were compatible with each outcome domain in each study were sought (e.g., for all measures, time points, analyses), and if not, the methods used to decide which results to collect.	pages 7 and 8
	10b	List and define all other variables for which data were sought (e.g., participant and intervention characteristics, funding sources). Describe any assumptions made about any missing or unclear information.	N/A
Study risk of bias assessment	11	Specify the methods used to assess risk of bias in the included studies, including details of the tool(s) used, how many reviewers assessed each study and whether they worked independently, and if applicable, details of automation tools used in the process.	page 8
Effect measures	12	Specify for each outcome the effect measure(s) (e.g., risk ratio, mean difference) used in the synthesis or presentation of results.	page 8
Synthesis methods	13a	Describe the processes used to decide which studies were eligible for each synthesis (e.g., tabulating the study intervention characteristics and comparing against the planned groups for each synthesis (item 5)).	N/A
	13b	Describe any methods required to prepare the data for presentation or synthesis, such as handling of missing summary statistics, or data conversions.	N/A
	13c	Describe any methods used to tabulate or visually display results of individual studies and syntheses.	N/A
	13d	Describe any methods used to synthesize results and provide a rationale for the choice(s). If meta-analysis was performed, describe the model(s), method(s) to identify the presence and extent of statistical heterogeneity, and software package(s) used.	N/A
	13e	Describe any methods used to explore possible causes of heterogeneity among study results (e.g., subgroup analysis, meta-regression).	N/A
	13f	Describe any sensitivity analyses conducted to assess robustness of the synthesized results.	N/A
Reporting bias assessment	14	Describe any methods used to assess risk of bias due to missing results in a synthesis (arising from reporting biases).	N/A
Certainty assessment	15	Describe any methods used to assess certainty (or confidence) in the body of evidence for an outcome.	N/A
RESULTS			
Study selection	16a	Describe the results of the search and selection process, from the number of records identified in the search to the number of studies included in the review, ideally using a flow diagram.	pages 9–11
	16b	Cite studies that might appear to meet the inclusion criteria, but which were excluded, and explain why they were excluded.	N/A
Study characteristics	17	Cite each included study and present its characteristics.	pages 12–23
Risk of bias in studies	18	Present assessments of risk of bias for each included study.	page 10
Results of individual studies	19	For all outcomes, present, for each study: (a) summary statistics for each group (where appropriate) and (b) an effect estimate and its precision (e.g., confidence/credible interval), ideally using structured tables or plots.	pages 12–23
Results of syntheses	20a	For each synthesis, briefly summarize the characteristics and risk of bias among contributing studies.	N/A
	20b	Present results of all statistical syntheses conducted. If meta-analysis was conducted, present for each the summary estimate and its precision (e.g., confidence/credible interval) and measures of statistical heterogeneity. If comparing groups, describe the direction of the effect.	N/A
	20c	Present results of all investigations of possible causes of heterogeneity among study results.	N/A
	20d	Present results of all sensitivity analyses conducted to assess the robustness of the synthesized results.	N/A
Reporting biases	21	Present assessments of risk of bias due to missing results (arising from reporting biases) for each synthesis assessed.	N/A
Certainty of evidence	22	Present assessments of certainty (or confidence) in the body of evidence for each outcome assessed.	N/A
DISCUSSION			
Discussion	23a	Provide a general interpretation of the results in the context of other evidence.	pages 12–23
	23b	Discuss any limitations of the evidence included in the review.	pages 12–23
	23c	Discuss any limitations of the review processes used.	N/A
	23d	Discuss implications of the results for practice, policy, and future research.	pages 24–26
OTHER INFORMATION			
Registration and protocol	24a	Provide registration information for the review, including register name and registration number, or state that the review was not registered.	N/A
	24b	Indicate where the review protocol can be accessed, or state that a protocol was not prepared.	N/A
	24c	Describe and explain any amendments to information provided at registration or in the protocol.	N/A
Support	25	Describe sources of financial or non-financial support for the review, and the role of the funders or sponsors in the review.	N/A
Competing interests	26	Declare any competing interests of review authors.	N/A
Availability of data, code, and other materials	27	Report which of the following are publicly available and where they can be found: template data collection forms; data extracted from included studies; data used for all analyses; analytic code; any other materials used in the review.	N/A

**Table 2:** Search strategy used for the PubMed database.

Database	PubMed
Date	from inception to 22 December 2022
#1	“lung cancer epigenetic” AND ((DNA methylation diagnosis) OR miRNA diagnosis) OR lncRNA diagnosis) OR (liquid biopsy AND lung cancer diagnosis)) OR exhaled breath condensate) OR methylation detection methods) OR DNA methylation prognosis) OR miRNA prognosis) OR lncRNA prognosis) OR epigenetic-targeted therapy) OR novel therapeutics) OR clinical trials) OR preclinical trials) OR nutriceuticals) AND full text[sb] AND Humans[MeSH] AND English[lang])
#2	(((NSCLC diagnosis) AND (DNA methylation)) OR ((miRNA) AND (NSCLC diagnosis)) OR ((lncRNA) AND (NSCLC diagnosis)) OR ((liquid biopsy) AND (NSCLC diagnosis)) OR ((exhaled breath condensate) AND (NSCLC diagnosis)) AND full text[sb] AND AND Humans[MeSH] AND English[lang])
#3	(((NSCLC prognosis) AND (DNA methylation)) OR ((miRNA) AND (NSCLC prognosis)) OR ((lncRNA) AND (NSCLC prognosis)) AND full text[sb] AND Humans[MeSH] AND English[lang])
#4	(((NSCLC therapy) AND (DNA methylation)) OR ((miRNA) AND (NSCLC therapy)) OR ((lncRNA) AND (NSCLC therapy)) OR ((epigenetic therapy) AND (lung cancer)) OR ((novel therapeutics) AND (NSCLC)) OR ((epigenetic therapy) AND (clinical trial)) OR ((epigenetic therapy) AND (preclinical trial)) OR ((epigenetic therapy) AND (nutriceuticals)) AND full text[sb] AND Humans[MeSH] AND English[lang])

**Table 3:** The search items used for the systematic review.

Search Terms Used in the Systematic Review	
Lung cancer epigenetics	DNA methylation diagnosis
NSCLC diagnosis	miRNA diagnosis
NSCLC prognosis	lncRNA diagnosis
NSCLC therapy	Epigenetic therapy
Liquid biopsy	miRNA prognosis
Lung cancer diagnosis	lncRNA prognosis
Exhaled breath condensate	Epigenetic-targeted therapy
Methylation detection methods	Novel therapeutics
DNA methylation prognosis	Nutriceuticals

**Table 4:** Measures and methods for the studies included in the systematic review. AUC: area under curve.

Measures and Methods for the Studies Included in the Systematic Review		
Chapter	Measures	Methods
Section 4.1	Diagnostic epigenetic biomarkers in NSCLC	
	Cancerous vs. non-cancerous tissue	Sensitivity and specificity as given in AUC measures
Section 4.1.1	Exhaled breath condensate findings	Proportion in study population
Section 4.1.2	Bronchial secretions	Sensitivity and specificity as given in AUC measures
Section 4.1.3	Peripheral blood	Sensitivity and specificity
Section 4.1.4	Exosomes for detection of NSCLC	Size and concentration
Section 4.2	Prognostic epigenetic biomarkers in NSCLC	
	Classical survival parameters since diagnosis	Overall survival
Section 4.2.1	Single-gene/genome-wide DNA methylation profiling	Overall survival
Section 4.2.2	Non-coding RNA expression profiling	Survival parameters
Section 4.3	Epigenetic-based therapy for NSCLC	
	Efficacy of/response to treatment	Measurement of tumor load
Section 4.3.1	Natural substances and their derivatives	Expression vs. tumor load/apoptosis/growth/metastasis
Section 4.3.2	Synthetic epigenetic modalities	Inhibition of tumor cell growth and metastases

**Table 5:** Qualitative survival results of single-gene/genome-wide DNA methylation profiling.

Results of Single-Gene/Genome-Wide DNA Methylation Profiling	
Methylation	Result
DAPK1 [29] and TUSC3 [30] in NSCLC	Improved overall survival
P16/INK4a and BRCA1 [31] in adenocarcinoma	Shorter overall survival
RARβ [31] in adenocarcinoma	Longer overall survival
HOXA2 and HOXA10 [32] in squamous cell carcinoma	Shorter overall survival
HOXA9 in NSCLC lifelong non-smokers [33]	Poor recurrence-free survival
NPTX1 in NSCLC [34]	Shorter overall survival
PTPRH in adenocarcinoma [35]	Poor prognosis (OS)
AGTRL, ALDH1A3, BDKRB1, CTSE, EFNA2, NFAM1, SEMA4A, and TMEM129 in adenocarcinoma [36]	Poor prognosis (OS)

## Data Availability

The datasets analyzed during the current study are available from the corresponding author on reasonable request.
